# Anesthetic Management of a Tracheal Deformity in an Elderly Patient With Chronic Obstructive Pulmonary Disease: A Case Report

**DOI:** 10.7759/cureus.102432

**Published:** 2026-01-27

**Authors:** Kayo Hirose

**Affiliations:** 1 Department of Anesthesiology and Pain Relief Center, The University of Tokyo Hospital, Tokyo, JPN; 2 Faculty of Nutrition, Tokyo Kasei University, Tokyo, JPN

**Keywords:** airway management, chronic obstructive pulmonary disease, computed tomography, general anesthesia, saber sheath trachea

## Abstract

Saber sheath trachea is a tracheal deformity characterized by marked transverse narrowing relative to the anteroposterior diameter and is frequently associated with chronic obstructive pulmonary disease (COPD). Although saber sheath trachea has been discussed as a potentially challenging airway in anesthetic practice, perioperative airway complications have been reported mainly in individual case reports, and their true incidence, severity, and clinical implications remain uncertain.

We report the anesthetic management of an elderly patient with COPD and saber sheath trachea undergoing laparoscopic surgery under general anesthesia. Preoperative chest computed tomography demonstrated a marked transverse narrowing of the intrathoracic trachea, and quantitative assessment of tracheal dimensions was performed. Based on these findings, the endotracheal tube was selected with a careful consideration of its external diameter. Airway management and extubation strategies were planned in advance, with particular attention to perioperative phases associated with increased airway vulnerability.

General anesthesia was induced and maintained uneventfully, and intraoperative ventilation remained stable without cuff leakage or ventilatory instability. Extubation was performed cautiously with preparation for potential airway obstruction. No perioperative airway complications, including negative pressure pulmonary edema, were observed, and the postoperative course was uncomplicated.

This case illustrates that saber sheath trachea should be interpreted as a morphological condition rather than an automatic high-risk airway label. In patients with predominant transverse narrowing, CT-based assessment of tracheal dimensions may help guide airway planning, particularly with respect to endotracheal tube selection and extubation strategy.

## Introduction

Saber sheath trachea is a tracheal deformity first proposed in 1975, characterized by marked narrowing of the transverse tracheal diameter relative to the anteroposterior diameter on chest computed tomography (CT) [1]. It is commonly assessed using the ratio of the transverse to anteroposterior diameter, known as the tracheal index, which is typically measured at the level of the thoracic aortic arch. Although the diagnostic cutoff varies among reports, a tracheal index less than approximately 0.67 is most commonly used [1-4]. Although saber sheath trachea is typically identifiable on chest CT, this radiologic sign has been reported to be under-recognized in routine clinical practice [5]. This under-recognition may be attributable to heterogeneity in diagnostic criteria, including differences in measurement level and cutoff values for the tracheal index, rather than an accurate reflection of its true incidence.

This condition is thought to result from repetitive mechanical stress on the tracheal cartilage due to chronic coughing and significant fluctuations in intrathoracic pressure, which are hallmarks of severe chronic obstructive pulmonary disease [6]. Saber sheath trachea represents a static anatomical deformity identifiable on chest CT. Although anesthetic concerns such as cuff leakage and airway instability have been discussed in the literature, these issues are more typically associated with dynamic airway collapse rather than with the static morphology of saber sheath trachea itself. Therefore, it is important to distinguish saber sheath trachea from dynamic airway collapse, as the latter involves expiratory airway instability and carries different anesthetic implications.

Perioperative airway complications related to saber sheath trachea have been described primarily in isolated case reports, and their incidence, severity, and clinical implications remain insufficiently characterized. Nevertheless, these reports indicate that clinically significant ventilatory compromise, including airway obstruction and negative pressure pulmonary edema, can occur under certain conditions [7,8].

We report a case of general anesthesia in an elderly patient with COPD and saber sheath trachea, focusing on how preoperative CT-based anatomical assessment informed airway management decisions rather than reliance on the diagnostic label alone.

## Case presentation

The patient is a 71-year-old man with a height of 162.1 cm and a weight of 74.1 kg. His medical history included hypertension, hyperlipidemia, chronic obstructive pulmonary disease (COPD), and asthma, and he had been receiving inhaled therapy since the age of 70. He had a 46-pack-year smoking history and had quit smoking three years earlier. Formal spirometric data were not available at the time of surgery, and pulmonary function was evaluated clinically based on symptoms, physical findings, and imaging. Laparoscopic cholecystectomy was scheduled for gallstone disease. His preoperative respiratory status was stable, with no dyspnea or wheezing at rest. No apparent exacerbation of respiratory symptoms was observed during the preoperative evaluation.

**Figure 1:**
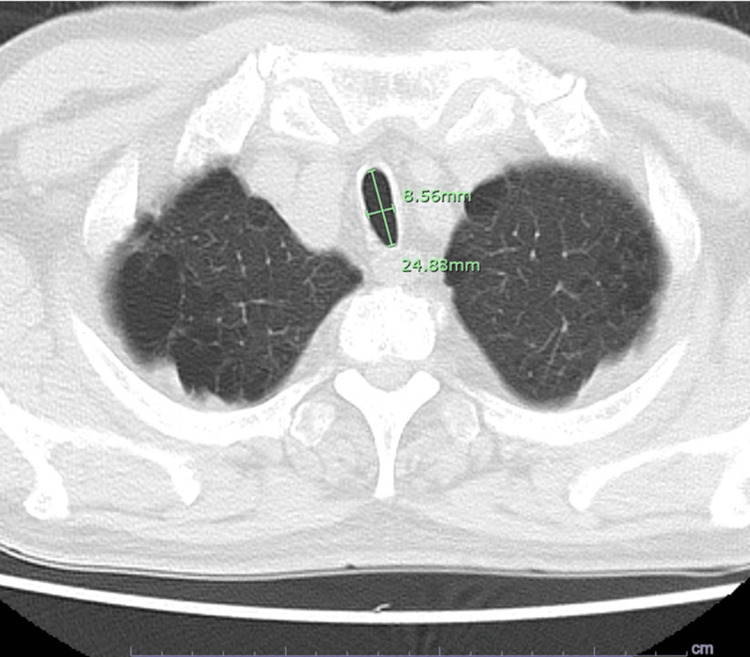
Axial chest computed tomography demonstrating saber-sheath trachea. Axial chest computed tomography image demonstrating marked transverse narrowing with relative anteroposterior widening of the trachea, consistent with saber-sheath trachea. At the narrowest segment, the transverse diameter was 8.56 mm and the anteroposterior diameter was 24.8 mm (tracheal index, 0.34).

A preoperative chest radiograph revealed tracheal narrowing with a transverse diameter of approximately 7 mm extending over a length of about 35 mm. Chest computed tomography (CT) demonstrated marked transverse narrowing of the trachea. The tracheal index is typically assessed at the level of the thoracic aortic arch. In this patient, a similar morphology was observed at that level, and the tracheal index was consistent with saber-sheath trachea. The narrowest segment was located at the level of the second thoracic vertebra, where the transverse and anteroposterior diameters measured 8.56 mm and 24.8 mm, respectively, yielding a tracheal index of 0.34. These measurements were used primarily for endotracheal tube size selection and perioperative planning (Figure 1).

**Figure 2:**
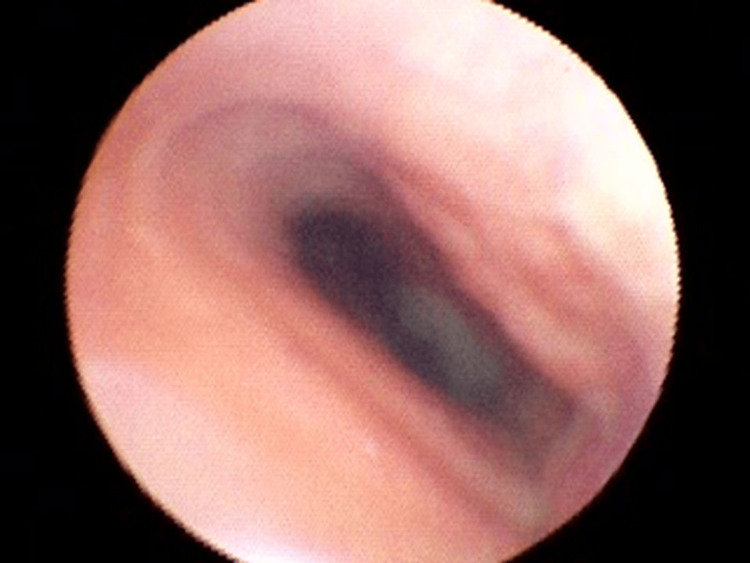
Bronchoscopic view of the trachea showing elliptical luminal narrowing. Fiberoptic bronchoscopic view obtained after induction of anesthesia showing an elliptical narrowing of the tracheal lumen corresponding to the stenotic segment identified on preoperative computed tomography, without inflammatory or neoplastic mucosal changes.

General anesthesia was induced with propofol, remifentanil, and fentanyl, and was maintained with propofol and remifentanil. Considering the transverse diameter of the narrowest tracheal segment measured on preoperative CT, an endotracheal tube with an internal diameter of 6.0 mm and an external diameter of 8.4 mm was selected. Considering the transverse diameter of the narrowest segment, an endotracheal tube with an external diameter of 8.4 mm was selected, and intubation was performed under bronchoscopic guidance to ensure safe passage and positioning. Because the margin between the tracheal transverse diameter and the tube outer diameter was limited, tracheal intubation was performed under fiberoptic bronchoscopic guidance to confirm the stenotic segment, assess resistance during advancement, and ensure that the cuff did not overlap the stenotic segment while maintaining an adequate depth. The cuff pressure was adjusted to 30 cmH₂O. Fiberoptic bronchoscopy performed after induction of anesthesia confirmed tracheal narrowing corresponding to the site identified on preoperative imaging. No apparent inflammatory changes or neoplastic lesions were observed in the tracheal mucosa (Figure 2).

During surgery, no cuff leakage was observed during changes in patient positioning or pneumoperitoneum, and ventilation remained stable.

Neuromuscular blockade was achieved with rocuronium and fully reversed with sugammadex prior to extubation. Mechanical ventilation was managed using volume-controlled ventilation with tidal volumes of approximately 6-8 mL/kg of predicted body weight with a positive end-expiratory pressure (PEEP) of 5 cmH₂O, with respiratory rate adjusted to maintain normocapnia. Extubation was performed cautiously after confirming full reversal of neuromuscular blockade and adequate spontaneous ventilation. Prior to extubation, airway patency and cuff position were reassessed bronchoscopically, and extubation was performed with preparation for immediate airway reassessment if required.

The operative time was two hours and nine minutes, the total anesthesia time was three hours and 19 minutes, and blood loss was 33 mL.

In anticipation of potential airway narrowing or tracheal collapse after extubation, fiberoptic bronchoscopy and reintubation equipment were prepared at the time of extubation. After the return of spontaneous breathing, a cuff leak test was performed and confirmed to be unremarkable, and the patient was extubated. Extubation was performed with the patient fully awake, following confirmation of adequate spontaneous ventilation, intact airway reflexes, and effective cough response. The patient was responsive to verbal commands, and no signs of respiratory distress were observed at the time of extubation. No dyspnea or airway obstruction occurred after extubation. Postoperative chest radiography showed no abnormalities, including negative pressure pulmonary edema. The postoperative course was uneventful, and the patient was discharged on postoperative day 4.

## Discussion

The key message of this case is that saber sheath trachea should be interpreted as a morphological condition that requires individualized assessment rather than automatic classification as a high-risk airway. Therefore, anesthesiologists should approach such patients with appropriate caution, ensuring thorough preoperative anatomical assessment and preparedness for prompt intervention if airway complications arise. Rather than implying safety based on the apparent rarity of reported events, this condition warrants careful perioperative planning and vigilance throughout airway management.

A tracheal index of 0.34 places the present case at the severe end of the spectrum of reported saber sheath trachea. A previous radiological study by Ciccarese et al. demonstrated that lower tracheal index values are associated with more advanced airflow obstruction and greater disease severity in patients with chronic obstructive pulmonary disease [3]. This degree of transverse narrowing, therefore, provided important quantitative context for risk assessment and directly motivated the conservative endotracheal tube selection strategy based on external diameter. In the present case, such preparedness was achieved primarily through detailed anatomical evaluation using preoperative chest computed tomography. The narrowest tracheal segment was identified, and both transverse and anteroposterior diameters were quantitatively assessed. Based on these measurements, the size of the endotracheal tube was selected after a careful consideration of its external diameter to avoid excessive airway resistance or malposition. Previous reports have emphasized that anesthesiologists should directly evaluate tracheal morphology on preoperative CT in patients with respiratory disease. [9] In this case, CT-based anatomical assessment proved valuable for anesthetic planning.

In contrast, reliance on plain chest radiography alone may be insufficient for accurate evaluation of tracheal anatomy in such patients [10]. It should be noted, however, that chest CT provides a static anatomical assessment and does not capture dynamic changes in tracheal caliber [8,11]. In this case, the respiratory phase during CT acquisition was not explicitly documented and therefore the measurements were interpreted as static reference values rather than as indicators of dynamic airway behavior. Accordingly, CT-based measurements were used primarily to inform endotracheal tube size selection and perioperative planning, rather than to predict airway collapse during spontaneous respiration.

Among the anesthetic concerns historically discussed in patients with saber sheath trachea, endotracheal tube cuff leakage has frequently been cited. However, cuff leakage has been reported in a patient with markedly elongated anteroposterior tracheal morphology, which differs from the typical transverse narrowing that characterizes classic saber sheath trachea [2]. To our knowledge, there are no published reports explicitly describing ventilatory failure due to cuff leakage in patients with saber sheath trachea characterized predominantly by transverse narrowing. However, this absence of reported cases should be interpreted cautiously, as it may reflect limited reporting or variability in diagnostic recognition rather than a true absence of risk. In the present case, transverse narrowing was the main anatomical feature, and ventilation remained stable throughout the procedure following endotracheal tube selection based on quantitative preoperative CT assessment. These observations suggest that cuff leakage may not represent a universal or predominant risk across all morphological subtypes of saber sheath trachea.

In elderly patients with chronic obstructive pulmonary disease, obstructive ventilatory impairment leads to increased functional residual capacity and residual volume, in addition to age-related respiratory muscle weakness and decreased chest wall compliance. This combination results in a “difficult-to-exhale” respiratory state [12,13]. Such respiratory mechanical changes tend to become more pronounced during forced expiration or conditions of increased ventilatory demand and may contribute to dynamic airway collapse and ventilatory impairment [11]. From this perspective, saber sheath trachea can be interpreted as a morphological manifestation of age-related and COPD-associated alterations in respiratory mechanics rather than an isolated anatomical abnormality.

Previous reports have described airway obstruction during emergence and extubation in selected patients with saber sheath trachea, suggesting that this phase may warrant particular attention despite an uneventful intraoperative course [14,15]. These reports highlight that the period surrounding extubation, when patients transition from controlled ventilation to spontaneous breathing, represents a particularly vulnerable phase. From a physiological perspective, the endotracheal tube may provide a temporary stenting effect that helps maintain airway patency across a narrowed tracheal segment during controlled ventilation. Following extubation, the loss of this stenting effect, combined with increased expiratory effort, coughing, or negative intrathoracic pressure, may predispose the trachea to dynamic narrowing or collapse, particularly in patients with COPD-related airflow limitation [11-15]. In the present case, extubation was therefore approached cautiously, with preparation of a fiberoptic bronchoscope and readiness for reintubation in anticipation of potential airway obstruction. Although no perioperative complications occurred, this structured approach was considered a reasonable preventive strategy informed by previously reported risks rather than a result of chance alone.

Based on the present case, a simple and practical approach to anesthetic management of saber sheath trachea identified preoperatively may be proposed. First, a careful measurement of the narrowest transverse tracheal diameter on chest CT allows an objective assessment of airway morphology. Second, selection of the endotracheal tube should prioritize the outer diameter rather than the internal diameter to ensure safe passage through the narrowed segment. Third, bronchoscopic guidance during intubation permits the direct visualization of the stenotic area, confirmation of tube position, and avoidance of cuff placement across the narrowed segment. Finally, extubation should be regarded as a high-risk phase, with anticipatory planning and readiness for immediate airway intervention. This stepwise strategy emphasizes anatomical reality and perioperative physiology over reliance on the diagnostic label alone.

Saber sheath trachea should not be assumed to invariably require overly invasive airway management or circulatory support. This condition is often incidentally identified on chest CT, and a substantial number of patients may undergo general anesthesia without a preoperative diagnosis. Because saber sheath trachea is often detected incidentally on imaging and may go unrecognized during routine preoperative evaluation, some patients may undergo general anesthesia without this diagnosis being documented. As a result, perioperative airway complications in such cases may not be systematically attributed to the underlying tracheal morphology [9,10]. However, whether this leads to underreporting of anesthesia-related airway complications, or whether such complications are genuinely infrequent, cannot be determined from the currently available data.

This case does not imply that airway complications can be reliably predicted based on CT findings alone. In particular, the lack of dynamic airway evaluation and uncertainty regarding the respiratory phase during CT acquisition limit the ability to assess expiratory tracheal collapse or airway behavior during spontaneous breathing. These limitations are inherent to static imaging and to the nature of a single case report. However, it illustrates how anatomical interpretation may assist anesthesiologists in balancing airway risk without assuming either inevitable complications or complete safety.

This approach may help anesthesiologists avoid both underestimation and overestimation of airway risk in patients with suspected tracheal deformities.

This case is reported in accordance with the CARE (CAse REport) guidelines for case reports.

## Conclusions

In this case, planned anesthetic management informed by preoperative chest CT evaluation of tracheal morphology was implemented in an elderly patient with COPD and saber sheath trachea, and general anesthesia was completed without perioperative airway complications.

Saber-sheath trachea has been associated with potential challenges in airway management, including the risk of ventilatory compromise during periods of increased airway vulnerability, such as extubation. Therefore, anesthesiologists should approach such patients with appropriate caution, ensuring thorough preoperative anatomical assessment and preparedness for prompt intervention should airway complications occur. In the present case, endotracheal tube selection based on external diameter and careful planning of the extubation strategy were guided by the patient’s individual tracheal morphology rather than by the disease label alone, reflecting a stepwise, anatomy-driven approach to airway management.

In conclusion, this case suggests that CT-based interpretation of tracheal morphology may offer practical information to support airway planning in selected patients with saber sheath trachea, particularly during extubation.
